# The Antidiabetic Agent Sodium Tungstate Induces Abnormal Glycogen Accumulation in Renal Proximal Tubules from Diabetic IRS2-Knockout Mice

**DOI:** 10.1155/2018/5697970

**Published:** 2018-05-29

**Authors:** Romina Bertinat, Francisco Westermeier, Pamela Silva, Rodrigo Gatica, Joana Moitinho Oliveira, Francisco Nualart, Ramón Gomis, Alejandro J. Yáñez

**Affiliations:** ^1^Centro de Microscopía Avanzada (CMA BIO-BIO), Universidad de Concepción, Concepción, Chile; ^2^Institute of Biomedical Science, FH Joanneum Gesellschaft mbH University of Applied Sciences, Eggenberger Allee 13, 8020 Graz, Austria; ^3^Facultad de Ciencia, Universidad San Sebastián, Santiago, Chile; ^4^Facultad de Salud, Universidad Santo Tomás, Osorno, Chile; ^5^Escuela de Veterinaria, Facultad de Ciencias, Universidad Mayor, Santiago, Chile; ^6^CIBER de Diabetes y Enfermedades Metabólicas Asociadas (CIBERDEM), Barcelona, Spain; ^7^Diabetes and Obesity Research Laboratory, IDIBAPS, Barcelona, Spain; ^8^Department of Endocrinology and Nutrition, Hospital Clinic, Barcelona, Spain; ^9^Faculty of Medicine, University of Barcelona, Barcelona, Spain; ^10^Facultad de Ciencias, Universidad Austral de Chile, Valdivia, Chile

## Abstract

The kidney is an insulin-sensitive organ involved in glucose homeostasis. One major effect of insulin is to induce glycogen storage in the liver and muscle. However, no significant glycogen stores are detected in normal kidneys, but diabetic subjects present a characteristic renal histopathological feature resulting from extensive glycogen deposition mostly in nonproximal tubules. The mechanism of renal glycogen accumulation is yet poorly understood. Here, we studied in situ glycogen accumulation in the kidney from diabetic IRS2-knockout mice and the effect of the insulin-mimetic agent sodium tungstate (NaW). IRS2-knockout mice displayed hyperglycemia and hyperinsulinemia. NaW only normalized glycemia. There was no evident morphological difference between kidneys from untreated wild-type (WT), NaW-treated WT, and untreated IRS2-knockout mice. However, NaW-treated IRS2-knockout mice showed tubular alterations resembling clear cells in the cortex, but not in the outer medulla, that were correlated with glycogen accumulation. Immunohistochemical detection of the gluconeogenic enzyme phosphoenolpyruvate carboxykinase, mostly expressed by renal proximal tubules, showed that altered tubules were of proximal origin. Our preliminary study suggests that IRS2 differentially regulates glycogen accumulation in renal tubules and that NaW treatment in the context of IRS2 ablation induces abnormal glycogen accumulation in cortical proximal tubules.

## 1. Introduction

Insulin participates in a wide spectrum of growth and metabolic responses by binding to the insulin receptor (IR) and inducing IR tyrosine kinase activity to phosphorylate IR itself and several other proteins, among them IR substrates (IRS) [1]. Knockout (KO) studies have been critical to understand IRS relevance in insulin signaling in different tissues, indicating that IRS1 plays a major role in adipose tissue and skeletal muscle metabolism [1–3], whereas IRS2 is critical for liver metabolism and pancreatic *β*-cell development and survival [1–4]. Moreover, IRS1 and IRS2 show differential relevance in mitogenic and metabolic insulin effects, respectively, as IRS1-KO mice are 50% smaller in size and insulin resistant but maintain normal glucose tolerance due to compensatory *β*-cell hyperplasia [3], whereas IRS2-KO mice develop type 2 diabetes due to *β*-cell failure [4] and decreased suppression of endogenous glucose production and hepatic glycogen synthesis [2].

Two key effects of insulin are its ability to induce glycogen synthesis in the liver and muscle and to suppress gluconeogenesis in the liver [1]. Indeed, insulin inhibits the expression of rate-limiting enzymes of gluconeogenesis, that is, phosphoenolpyruvate carboxykinase (PEPCK), fructose 1,6-bisphosphatase (FBPase), and glucose 6-phosphatase (G6Pase), whereas it activates the key enzyme of glycogen synthesis, that is, glycogen synthase (GS) [1]. The kidney is also an insulin-sensitive organ involved in normal glucose homeostasis and enhanced gluconeogenesis during diabetes [1, 5, 6]. However, contrary to the liver and muscle, no significant glycogen stores are detected in the kidney under normal conditions, but diabetes induces abnormal glycogen deposition especially in nonproximal tubular epithelial cells [7–10]. This pathological feature has long been recognized, but the triggering mechanisms are not fully understood, albeit overexpression and activation of GS may be involved [8, 9].

Sodium tungstate (NaW) is an inorganic salt that exerts potent insulin-mimetic and antidiabetic actions in animal models of diabetes by normalizing hepatic glycogen metabolism and blood glucose levels [11]. NaW is a phosphatase inhibitor [11–13], but this property cannot account for all its antidiabetic effects [13]. Notably, NaW stimulates hepatic glycogen synthesis bypassing IR [13, 14], with the common intracellular target of all NaW insulin-mimetic effects reported to date being the mitogen-activated protein kinase (MAPK) pathway, through phosphorylation/activation of extracellular signal-regulated kinase (ERK) 1/2, both *in vitro* and *in vivo* [11]. NaW has been shown to reverse the pathological accumulation of glycogen in the diabetic kidney [15], but it remains to be established whether NaW requires an intact insulin signaling pathway to exert its renal effects. In parallel, the adverse effects of NaW treatment are poorly defined, which has hampered further clinical study of this antidiabetic agent [11, 16].

The molecular pathways involved in the opposite response between the liver/muscle and kidney in glycogen accumulation during diabetes are poorly understood. On the basis that IRS2 is critical for the proper regulation of glycogen metabolism in the liver [2, 4], we hypothesized that it also has a pivotal role in the kidney, especially because IRS2 mRNA expression is higher in mouse kidney than in the liver [17]. Therefore, in this preliminary work, we examined the effect of IRS2 ablation and the impact of NaW treatment on glycogen accumulation in the kidney in situ. To this end, we used a total IRS2-KO mouse model that exhibits a diabetic phenotype [2, 4]. Our data suggest that IRS2 plays an important role in renal glycogen metabolism and that NaW treatment in the context of IRS2 ablation is harmful for the kidney.

## 2. Materials and Methods

### 2.1. Animals

Wild-type (WT) and whole-body IRS2-KO mice were generated by intercrossing Irs2^+/−^ mice (C57Bl/6 genetic background) [4]. The principles of laboratory animal care were followed (European and local government guidelines), and protocols were approved by the Animal Research Committee of Barcelona University. 10-week-old male WT and IRS2-KO mice with glycemia inferior to 16 mM were randomly divided into two groups. For 3 weeks, one WT group received distilled water as drinking water (untreated group), whereas the other received ad libitum a solution of 2 mg/ml NaW (Sigma-Aldrich, St. Louis, MO) in distilled water (treated group). The same is true for the two IRS2-KO groups. Glycemia was measured with a clinical glucometer. Insulinemia was analyzed with an ELISA kit (Mercodia, Uppsala, Sweden). Animals were euthanized by cervical dislocation.

### 2.2. Antibodies

Rabbit anti-cytosolic PEPCK was prepared in our laboratory [18]. Goat anti-muscle pyruvate kinase (PK-M) was from Rockland Immunochemicals Inc. (Gilbertsville, PA). An HRP-conjugated *β*-actin antibody was from Santa Cruz Biotechnology (sc-47778-HRP; Santa Cruz, CA). Rabbit monoclonal anti-phospho-p44/42 MAPK (Erk1/2) (Thr202/Tyr204) was from Cell Signaling Technology Inc. (#4370, Beverly, MA). Secondary antibodies were goat anti-rabbit IgG-HRP, rabbit anti-goat IgG-HRP (Jackson ImmunoResearch, West Grove, PA), and Alexa Fluor 488-conjugated donkey anti-rabbit IgG (Molecular Probes, Eugene, OR).

### 2.3. Histochemical Analysis

Pieces of the kidney were fixed in formalin and embedded in paraffin. Immunohistochemical studies were conducted as previously described [18]. Briefly, tissue was deparaffinized in xylene, rehydrated in graded ethanol, blocked with 3% BSA, and permeabilized with 0.05% Triton X-100 for 20 min. For immunoperoxidase and immunofluorescence analyses, primary antibodies were incubated overnight at 4°C and secondary antibodies were incubated for 1 h at room temperature. For periodic acid-Schiff (PAS) staining, we used the PAS Staining Kit from Merck Millipore (Billerica, MA), following the manufacturer's instructions. In situ digestion of glycogen was performed with *α*-amylase (Sigma-Aldrich, St. Louis, MO; 0.2% *w*/*v α*-amylase in 0.1 M Na_2_HPO_4_ and 0.05 M sodium citrate, pH 5.0) for 30 min at 37°C in a humidified chamber. Serial sections of the kidney were mounted on separate slides: one was digested and the other was not, but both were stained with PAS reagents. For semiquantitation of PAS staining, images were smoothed by applying a 3-pixel Gaussian filter. Areas of interest with higher PAS signal, before and after *α*-amylase treatment, were manually selected using the Wand tracing tool from ImageJ v1.48 software (NIH, USA), and the total area was calculated. Calculation of the total tissue area was based on the selection of all pixels with saturation higher than 30. For combined PAS staining and immunofluorescence, we applied a previously described protocol where tandem detection was standardized [19], using DAPI for nuclear counter staining. ImageJ v1.48 software (NIH, USA) was used to digitally combine images from immunofluorescence and bright-field PAS staining.

### 2.4. Western Blot

Proteins were prepared in lysis buffer (50 mM Tris pH 7.5, 5 mM EDTA, 150 mM NaCl, 1% Triton X-100, 10 mM sodium phosphate, 10 mM sodium fluoride, 10 mM sodium orthovanadate, and protease inhibitors), resolved by 10–12% SDS-PAGE, transferred onto PVDF membranes, and probed overnight with a primary antibody. *β*-Actin was used as a loading control. Reaction was developed using the Pierce ECL Western Blotting Substrate (Pierce Biotechnology, Rockford, IL).

### 2.5. Statistical Analysis

Data are expressed as mean ± SD. Statistical comparison between groups was performed with 2-way ANOVA (followed by Tukey's post hoc test) using GraphPad Prism software, version 6.01. Data were considered statistically significant for *P* < 0.05.

## 3. Results

**Figure 1:**
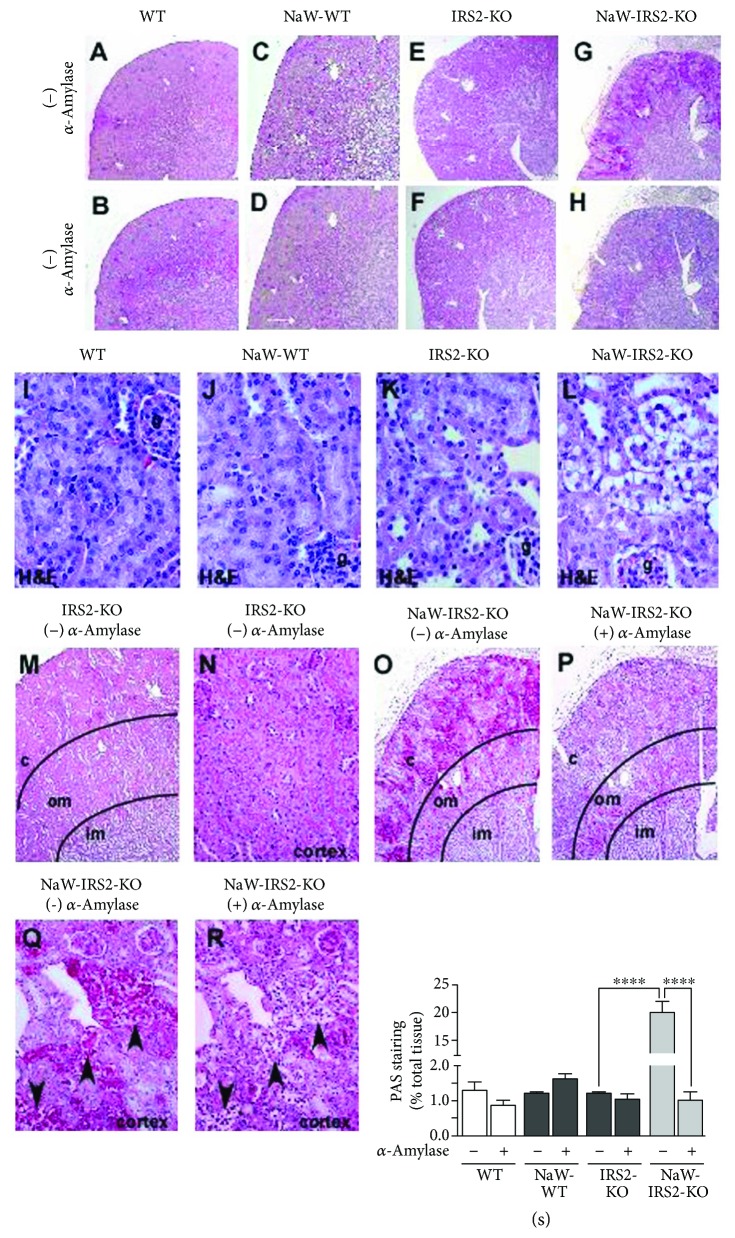
NaW induces glycogen accumulation in renal cortical tubules from IRS2-KO mice. Serial sections of the kidney from untreated WT (WT; a, b), treated WT (NaW-WT; c, d), untreated IRS2-KO (IRS2-KO; e, f), and treated IRS2-KO (NaW-IRS2-KO; g, h) mice were processed for PAS staining with (+) or without (−) *α*-amylase digestion. (i–l) Hematoxylin/eosin (H&E) staining to compare renal morphology. (m) Magnification of (e). (n) Magnification of (m). (o) Magnification of (g). (p) Magnification of (h). (q) Magnification of (g) and (o). (r) Magnification of (h) and (p). Areas of positive PAS staining were semiquantified and normalized against total tissues. c: cortex; om: outer medulla; im: inner medulla. Arrowheads show the same tubules in serial renal sections before and after *α*-amylase digestion. Representative images of *n* = 5 each group. ^∗∗∗∗^*P* < 0.0001.

Ten-week-old IRS2-KO mice displayed fasting hyperglycemia and hyperinsulinemia. Three weeks of NaW treatment had no effect on control WT mice and was able to only normalize glycemia but not insulinemia in IRS2-KO mice. Body weight was not significantly affected by the treatment (Table 1). Noteworthy, IRS2-KO mice did not display polydipsia at this stage yet; therefore, both control and IRS2-KO mice ingested almost the same amount of water and consumed almost the same amount of NaW, which was approximately 120 mg per kg body weight per day. In order to complement the morphological data and extend the study of IRS2 and NaW participation in renal insulin signaling from IRS2-KO mice, we conducted PAS staining with or without *α*-amylase digestion to asses for glycogen deposition in situ. Unexpectedly, no differences were detected between untreated WT (WT), treated WT (NaW-WT), and untreated IRS2-KO (IRS2-KO) kidneys with or without *α*-amylase digestion (Figures 1(a)–1(f), resp.), indicating that no significant glycogen stores were present in these conditions, despite hyperglycemia in IRS2-KO mice. In contrast, the same analysis in kidneys from NaW-treated IRS2-KO (NaW-IRS2-KO) mice showed a significant increase in PAS staining (Figure 1(g)) that was *α*-amylase-sensitive (Figure 1(h)). Hematoxylin/eosin staining confirmed that no significant differences in kidney morphology were evident between WT, NaW-WT, and IRS2-KO mice (Figures 1(i)–1(k), resp.), but kidneys from NaW-IRS2-KO mice showed a distinctive pattern of tubular morphology (Figure 1(l)). A more detailed comparison of kidneys from IRS2-KO (Figures 1(m) and 1(n)) and NaW-IRS2-KO (Figures 1(o)–1(r)) mice at higher magnification showed that increased *α*-amylase-sensitive PAS-positive tubules were mainly localized to the renal cortex and not to the outer medulla from NaW-IRS2-KO mice (Figures 1(o) and 1(p)). Semiquantitation of those renal areas that were positive for PAS staining and sensitive to *α*-amylase revealed that approximately 20% of the renal cortex presented an increased signal in NaW-IRS2-KO mice that was the result of glycogen accumulation (Figure 1(s)).

**Figure 2:**
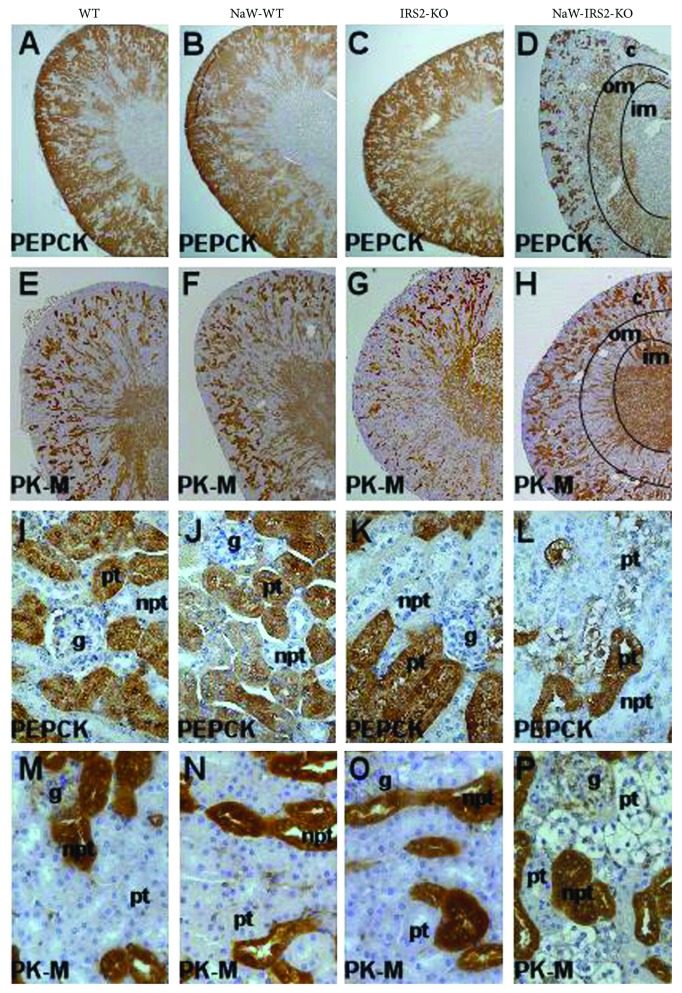
Zonation of metabolic enzymes in the kidney from NaW-IRS2-KO mice. The gluconeogenic enzyme PEPCK (a–d) and the glycolytic enzyme PK-M (e–h) were immunodetected in formalin-fixed paraffin-embedded kidneys from untreated WT (WT), treated WT (NaW-WT), untreated IRS2-KO (IRS2-KO), and treated IRS2-KO (NaW-IRS2-KO) mice. (i–l) Magnifications of the renal cortex from (a)–(d), respectively. (m–p) Magnifications of the renal cortex from (e)–(h), respectively. c: cortex; om: outer medulla; im: inner medulla, pt: proximal tubule; npt: nonproximal tubule; g: glomerulus. Representative images of *n* = 5 each group.

To define which renal tubules were affected by glycogen accumulation in NaW-IRS2-KO mice, we analyzed the cellular expression of different metabolic enzymes that display a characteristic pattern of zonation along the nephron, which may help to understand the segment specificity (if any) of the glycogenic alteration. We studied the gluconeogenic enzyme PEPCK, which is specifically expressed in proximal tubules, and the muscle isoform of the glycolytic enzyme pyruvate kinase (PK-M) that is mostly expressed downstream of the proximal tubules. Given that tubular structures downstream of the proximal tubule have different functions and names, hereafter, we will refer to them just as nonproximal tubules for simplification. As expected, PEPCK was exclusively detected in the renal cortex and outer medulla from WT, NaW-WT, IRS2-KO, and NaW-IRS2-KO mice, where proximal tubules are organized (Figures 2(a)–2(d), resp.). Instead, PK-M was detected in the cortex and outer medulla and all tubule segments from the inner medulla in the same groups (Figures 2(e)–2(h)), in agreement with the distribution of nonproximal tubules throughout the tissue. However, in the particular case of NaW-IRS2-KO mice, PEPCK showed a different immunoreactivity in the renal cortex but not in the outer medulla (Figure 2(d)), whereas PK-M immunostaining remains virtually the same among the groups (Figures 2(e)–2(h)). Although PEPCK detection was limited to proximal tubules (Figures 2(i)–2(l)), its immunoreactivity was reduced in those tubules that show a different morphology in NaW-IRS2-KO kidneys, in comparison to tubules not affected in the same samples (Figure 2(l)) and to those in WT, NaW-WT, and IRS2-KO mice (Figures 2(i)–2(k), resp.). A similar pattern of immunoreactivity as for PEPCK was detected with an antibody against FBPase, another gluconeogenic enzyme expressed in proximal tubules (data not shown). Again, no significant changes in the immunostaining of PK-M were detected in any group (Figures 2(m)–2(p)). We provide more evidence supporting the fact that neither NaW treatment nor IRS2 ablation per se produced an evident impact on renal morphology, but instead we observed that NaW treatment in the context of IRS2 ablation generated a disarrangement of cortical glycogen metabolism, which was concentrated in proximal tubules.

**Figure 3:**
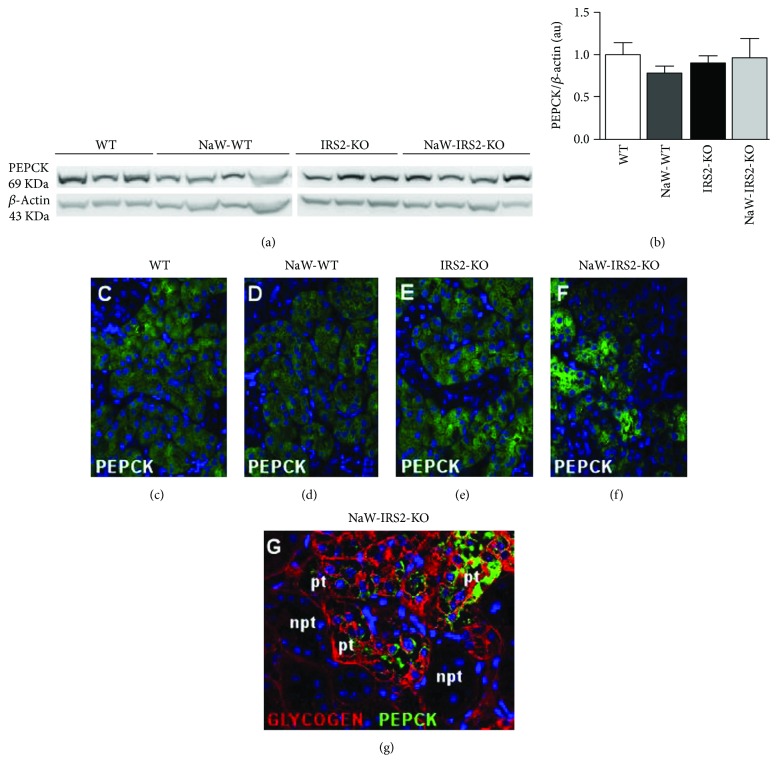
Protein expression of the gluconeogenic enzyme PEPCK in the kidney from NaW-IRS2-KO mice. Western blot analysis of PEPCK in the total kidney from untreated WT (WT), treated WT (NaW-WT), untreated IRS2-KO (IRS2-KO), and treated IRS2-KO (NaW-IRS2-KO) mice (a, b). (c–f) Immunofluorescence analysis of PEPCK in the cortex from WT, NaW-WT, IRS2-KO, and NaW-IRS2-KO kidneys. (g) Combined glycogen (red) and PEPCK (green) detection in kidneys from NaW-IRS2-KO mice without *α*-amylase treatment. DAPI was used for nuclear staining. pt: proximal tubule, npt: nonproximal tubule. Representative images of *n* = 5 each group.

Interestingly, despite that the immunohistochemical data showed an apparent reduction in the intensity of PEPCK immunostaining in the kidney cortex between NaW-IRS2-KO and the other groups (Figures 2(a)–2(d) and 2(i)–2(l)), Western blot analysis revealed no significant differences in the level of expression of PEPCK in whole kidney samples (Figures 3(a) and 3(b)). Given that immunoperoxidase analysis produced strong signal amplification, we performed immunofluorescence analysis to study PEPCK expression and distribution with more detail. Confocal analysis showed no significant differences among samples from WT, NaW-WT, and IRS2-KO mice (Figures 3(c)–3(e), resp.), but a great variability in PEPCK signal among proximal tubules from NaW-IRS2-KO mice was observed, with some proximal tubules showing enhanced reaction and others showing very poor levels (Figure 3(f)), which was further confirmed by a combination of PAS staining and PEPCK immunofluorescence (Figure 3(g)).

**Figure 4:**
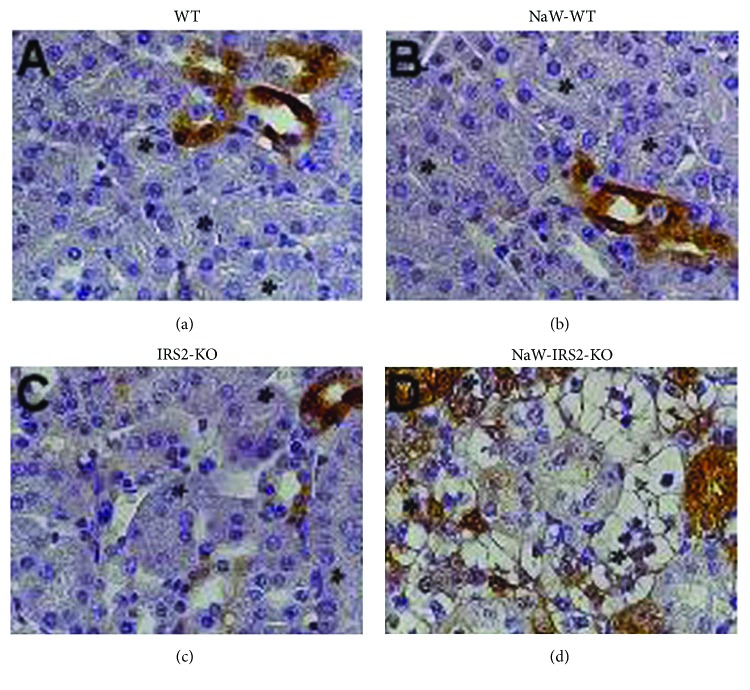
NaW-induced ERK1/2 activation in proximal tubules from the IRS2-KO kidney. Activation of ERK1/2 was analyzed in situ by immunodetection of phosphorylated residues Thr202/Tyr204 in formalin-fixed paraffin-embedded kidneys from (a) untreated WT (WT), (b) treated WT (NaW-WT), (c) untreated IRS2-KO (IRS2-KO), and (d) treated IRS2-KO (NaW-IRS2-KO) mice. Asterisks: proximal tubule. Representative images of *n* = 4 each group.

Given that one main intracellular target of NaW is ERK1/2, which has been shown to mediate NaW-dependent glycogen production in hepatocytes [13], we studied in situ phosphorylation of this signaling molecule to assess its role in renal glycogen accumulation in the IRS2-KO model. Interestingly, phosphorylation of ERK1/2 was detected in the kidney from both untreated WT and IRS2-KO mice, but only in nonproximal tubules (Figures 4(a) and 4(c), resp.). NaW treatment had no evident effect on further activation of ERK1/2 in the kidney from WT mice (Figure 4(b)), but instead it stimulated ERK1/2 phosphorylation in proximal tubules from IRS2-KO mice (Figure 4(d)).

## 4. Discussion

Despite the relevance of the kidney in glucose homeostasis and the well-known antidiabetic properties of NaW, only one study has addressed its effect on renal tissue, showing that glycogen accumulation in renal tubules, which is the most characteristic histopathological lesion related to the diabetic kidney, was absent in NaW-treated WT diabetic rats compared with the untreated diabetic group [15]. Given that NaW mimics most of insulin effects but acts through different downstream effectors, in the present work, we aimed to extend our understanding regarding its effects on renal glucose metabolism by using the diabetic IRS2-KO mouse model that shows reduced hepatic glycogen metabolism [2, 4]. On this basis, we hypothesized that this diabetic model is characterized by decreased glycogen accumulation in the liver and increased glycogen accumulation in the kidney. Interestingly, a previous report showed that IRS2 mRNA expression was higher in the mouse kidney than in the liver [17]. However, despite these differences, no significant morphological alterations were detected between kidneys from WT and IRS2-KO mice [4, 17, present study].

NaW treatment normalized glycemia but not insulinemia in IRS2-KO mice, in agreement with a previous work where improved glucose tolerance was not associated with enhanced peripheral insulin sensitivity, but rather with increased *β*-cell mass and replication and a reduction in *β*-cell apoptosis [4]. Indeed, IRS2-KO mice showed altered hepatic glucose metabolism, with lower glycogen levels and a marked decrease in the ability of insulin to suppress hepatic glucose production [2, 4], a phenotype that was not improved by NaW treatment [4]. Conversely, NaW has been previously shown to restore hepatic glycogen metabolism in other diabetic animal models with an intact insulin pathway [11], strongly suggesting that IRS2 is associated with hepatic insulin-mimetic effects of NaW.

Insulin signaling negatively modulates hepatic and renal gluconeogenesis by inhibiting expression of gluconeogenic enzymes [1, 5, 6], although renal regulation seems to differ from that of the liver [5]. The metabolic zonation of gluconeogenic enzymes in proximal tubules and glycolytic enzymes in nonproximal tubules is in agreement with the differential ability of each nephron portion to handle glucose [20, 21]. In the present work, we observed that PEPCK immunostaining was severely reduced only in those cortical proximal tubules affected by glycogenic alteration in NaW-IRS2-KO mice. It has been reported that NaW, as an insulin-mimetic agent, downregulates PEPCK expression in the liver from WT diabetic rats [22] and in human proximal tubules *in vitro* [23]. However, previous findings described an inconsistent effect of NaW in downregulating PEPCK in the liver from IRS2-KO mice [4], suggesting that IRS2 expression is required. Although further analysis is needed, we show that NaW is also unable to downregulate renal PEPCK expression in the context of IRS2 ablation, and therefore the preservation of PEPCK total protein levels in the kidney from NaW-IRS2-KO compared to IRS2-KO mice might be explained by overexpression in proximal tubules not affected, together with downregulation in proximal tubules affected by glycogen accumulation, which in combination may not be reflected in Western blot from the total kidney. Thus, the reduced expression of PEPCK may be the consequence of cell death after extensive glycogen accumulation [7, 9] rather than a direct downregulating effect exerted by NaW on the expression of gluconeogenic enzymes, as reduced immunodetection is only observed in proximal tubules that show severe glycogenic alterations.

One main and unexpected finding of our preliminary work is the absence of tubular alterations related to abnormal glycogen accumulation in diabetic IRS2-KO mice. We expected some level of glycogen deposition due to the diabetic phenotype. It has been shown that expression and/or activation of GS may be involved in abnormal glycogen accumulation of diabetic kidney [8, 9, 24]. GS is regulated by glucose 6-phosphate (G6P) allosteric activation and by inactivating phosphorylation [1, 8, 25]. Fully phosphorylated GS is considered to be inactive. Noteworthy, it has been described that glycogen-storing cells are mainly originated by nonproximal tubules in kidneys from WT diabetic rats [7–10] and that NaW treatment reverses this phenotype [15]. However, no glycogen-storing cells were detected in the kidney from diabetic IRS2-KO mice, indicating that hyperglycemia itself is not enough to promote renal glycogen synthesis. At this regard, it has been reported that adiponectin activates AMP-activated protein kinase (AMPK) in distal tubules and thick ascending limb to phosphorylate and inhibit GS, inhibiting in turn glycogen accumulation [8]. Although the involvement of IRS2 in the renal adiponectin-AMPK-GS axis remains to be elucidated, we detected activation of ERK1/2 in renal proximal tubules from IRS2-KO but not from WT mice after NaW treatment, strongly suggesting that this signaling molecule participates in NaW-induced glycogen production in IRS2-KO kidney. In agreement, ERK1/2 is pivotal for most insulin-mimetic effects of NaW, especially glycogen production in hepatocytes [11, 13]. Overstimulation of ERK1/2 by NaW in the absence of IRS2 may aberrantly activate glycogen synthesis, suggesting that insulin signaling through IRS2 keeps ERK1/2 activity restrained in proximal tubules to avoid glycogen production in WT mice.

Then, what is happening in the kidney from IRS2-KO mice after NaW treatment? Our preliminary data show that when IRS2 is absent and mice are treated with NaW, glycogen synthesis is activated in cortical proximal tubules, suggesting that IRS2 somehow inhibits glycogen accumulation in this particular tubular structure, possibly by keeping ERK1/2 inactive or at a low rate. During hyperglycemia, proximal tubules reabsorb more glucose from the glomerular ultrafiltrate [26]. However, it has been proposed that they just participate in transepithelial glucose movement but are not able to phosphorylate it into G6P, because proximal tubules virtually lack hexokinase activity [20, 21]. Then, reabsorbed luminal glucose cannot be directly phosphorylated into G6P, the actual precursor/activator of glycogen synthesis [1, 25]. Other possibility is indirect glycogen synthesis from G6P provided by gluconeogenesis, which is an important pathway in hepatic glycogen synthesis [27]. Moreover, NaW has been shown to inhibit G6Pase activity [12], the gluconeogenic enzyme that converts G6P into free glucose, but has no direct effect on FBPase activity [28]. Hence, during normal or enhanced gluconeogenesis in combination with reduced G6Pase activity, accumulation of G6P might be the triggering mechanism for glycogen accumulation, despite that GS is phosphorylated. Indeed, although phosphorylation leads to inactivation of GS, the activity can be restored in the presence of the allosteric activator G6P [25]. Thus, we speculate that NaW cannot inhibit gluconeogenesis in renal cortical proximal tubules in the absence of IRS2 that, together with NaW-induced G6Pase inhibition, favors G6P accumulation. This idea is sustained by the fact that gluconeogenic enzymes are not uniformly expressed along the proximal tubule, with higher levels in cortical proximal tubules [20] where we observed the glycogenic effect. Indeed, a recent report showed that targeted deletion of G6Pase in the kidney induces glycogen accumulation specifically in cortical proximal tubules [29], producing a pattern of tubular alteration similar to that observed in NaW-IRS2-KO mice. Accumulation of renal glycogen in the animal model studied by Clar et al. [29] confirmed its nephrotoxicity in the long term. While the effect of NaW on renal function in KO-IRS2 mice remains to be established, our histological data suggest that NaW-KO-IRS2 mice have nephropathy, although maybe incipient because of the short treatment compared with the 6 months of the experiment by Clar et al. [29].

NaW has been widely studied as an insulin-mimetic and antidiabetic drug because of its low hypoglycemic action, but other undesirable effects remain to be established [11]. The fact that NaW treatment affects glycogen metabolism in renal proximal tubules in IRS2-KO mice, similar to glycogen storage disease type 1a [29], turns on a red light on its potential use as a drug for type 2 diabetes treatment. Although IRS2 mutations are unusual and have not been associated with human type 2 diabetes, enhanced phosphorylation on serine/threonine residues, many of which negatively regulate IRS2 function [30], may affect the proper IRS2 downstream signals, which in turn would affect NaW renal effects. Indeed, only one study on human subjects has reached the clinical trial phase with inconclusive results [16], and a study with an organovanadium compound for the treatment of diabetes mellitus (with activity analogous to that of NaW) reached preclinical phase II but was terminated after 3 months because of renal complications (http://www.biospace.com/news/akesispharmaceuticalsdiscontinuessoleclinical/123583), indicating that the use of this kind of drugs might be specifically related to renal alterations *in vivo*. However, understanding the reasons behind clinical failure is essential for clinical development. Thus, a kidney-specific IRS2 deletion model would be critical to further understand the complex and particular roles of IRS2 in renal insulin signaling, glycogen metabolism, and NaW effects and also the participation of renal IRS2 in whole-body glucose homeostasis. In this sense, whole-body IRS2-KO mice showed impaired suppression of gluconeogenesis and reduced glycogen synthesis in the liver, but a liver-specific IRS2 deletion model showed that IRS2 is not required for glucose homeostasis, and rather extrahepatic IRS2-dependent mechanisms regulate this process [31].

## 5. Conclusions

In conclusion, our preliminary study showed two interesting and totally unexpected facts: (1) young diabetic IRS2-KO mice are protected against glycogen accumulation in renal tubules despite hyperglycemia and (2) NaW treatment normalized glycemia in IRS2-KO mice but induced glycogen accumulation in the kidney, specifically in proximal tubules from the cortex. Although more studies are needed, we propose that IRS2 is involved in pathological glycogen accumulation in the diabetic kidney and that the use of NaW in the context of altered IRS2 signaling induces abnormal renal glycogen accumulation that should be considered a new undesirable effect.

## Figures and Tables

**Table 1:** Blood glucose and insulin levels and body weight in groups of mice.

	WT	NaW-WT	IRS2-KO	NaW-IRS2-KO
Glycemia (mM)	7.6 ± 0.3	7.1 ± 0.2	13.0 ± 0.9^a^	7.0 ± 0.5^b^
Insulinemia (*μ*g/l)	1.5 ± 0.6	1.5 ± 0.7	2.1 ± 0.5^a^	2.0 ± 0.5
Body weight (g)	24.8 ± 3.5	25.5 ± 2.7	27.2 ± 1.3	24.0 ± 4.1

Values are means ± SD. *n* = 5 mice per group. ^a^*P* < 0.01 compared to WT. ^b^*P* < 0.001 compared to IRS2-KO.
